# Research agenda in family medicine—should we adopt the Dutch approach?

**DOI:** 10.1080/13814788.2019.1571733

**Published:** 2019-02-05

**Authors:** Shlomo Vinker, Mehmet Ungan

**Affiliations:** a EGPRN and WONCA Europe;; b Department of Family Medicine, Sackler Faculty of Medicine, Tel Aviv University, Tel Aviv, Israel;; c Department of Family Medicine, Ankara University School of Medicine, Ankara, Turkey

Burgers et al., in the current issue of the *European Journal of General Practice*, describe the approach they implemented to develop a research agenda for general practice in the Netherlands [1]. The impetus was the lack of coordination between organizations that fund research, and a vision to optimize filling gaps in clinical guidelines. Their approach was both ‘bottom-up’ and ‘top-down.’ They performed a systematic analysis of gaps in knowledge and practice by analysing Dutch clinical guidelines in family medicine, and attained input from stakeholders. Prioritizing was done at a later stage, mainly by practicing general practitioners. Hundreds of topics were prioritized by two dimensions: according to the International Classification for Primary Care (ICPC) domains and by common themes like ‘common conditions.’ Accordingly, the team built a roadmap for the most important topics for future research in family medicine in the Netherlands. This systematic and comprehensive work yielded an impressive list of research topics such as the effects of corticosteroid local injections, effective interventions for functional stomach complaints, complications of common infections and common reasons for constipation. These topics and many others can fill gaps of knowledge that are relevant to any primary healthcare system and that are usually not in the scope of hospital-based research. The most frequent research questions were in the domains of musculoskeletal problems, psychological problems, skin problems, and general and unspecific problems. These domains reflect the main clinical burden of family medicine, which has no substantial evidence in guidelines for primary care.

Notably, the long list of topics may be the antithesis to the free will and inspiration that drives researchers. While the Dutch roadmap enables coordinating research efforts and funding at a national level, it is not clear that it can be generalized to other countries and healthcare systems. We presume that each healthcare system should build its priority list using the same systematic approach.

We should ask ourselves a priori: do we need a dedicated research agenda for family medicine? If so, what steps are essential to increasing research in family medicine? In our opinion, a dedicated agenda is needed. Research generated by family physicians is practice as well as patient-centred; and, as such, reflects the core values of general practice. Building research capacity in family medicine is essential for ensuring that the evidence base for current clinical practice is relevant to the primary care setting and to the patients treated in this context. Further, such research is vital for enhancing the academic status and prestige of family medicine. Finally, from the physician’s point of view, it is an excellent way to reduce burnout, which is particularly acute among family physicians [2]. Involving trainees and young physicians in research projects may help to implement the role of family medicine in research and the academic world.

The Dutch approach compares to the request, almost one decade ago, by EGPRN of WONCA, to conceptualize a research agenda for family medicine. This yielded a series of six publications in this journal [3–8]. Similar to the Dutch approach, that initiative aimed to identify gaps in evidence, though with less comprehensive and diverse information sources. Assessment of effectiveness and efficiency are common to both agendas. However, the scope of the EGPRN-WONCA agenda addressed the use of different methodologies, contrasting with the Dutch approach in which methodology was not the main focus. For example, the EGPRN highlighted the importance of longitudinal cohort studies, intervention studies and randomized controlled trials. They stated that research should focus on diagnostic strategies and reasoning, and should also develop and validate functional and generic instruments and outcome measures for use in daily care.

We wholeheartedly agree with Burgers et al., that any research agenda should be continuously evaluated. This is particularly relevant due to the rapid changes, new technologies and blurring of boundaries between disciplines in medicine, which all characterize the current era. Both quantitative and qualitative research methods are important. Some of the questions that arise are: should we join big data research? Should artificial intelligence and decision support systems be part of our research agenda? Should we emphasize translational research and interdisciplinary research with other specialties? Alternatively, should we stress classical topics of family medicine such as understanding and clearly defining competencies, person-centeredness, comprehensiveness and a holistic approach, while developing research instruments and outcome measures for these competencies?

Certainly, one suit does not fit all, and healthcare systems should modify their research agenda according to their clinical and research capacities and interests [9]. The approach of the Dutch endeavour is important, in its calling for prioritization of healthcare topics. We suggest that more attention should be given to methodology and not only to the content of research.
